# Strong Genetic Structure Observed in *Primulina danxiaensis*, a Small Herb Endemic to Mount Danxia With Extremely Small Populations

**DOI:** 10.3389/fgene.2021.722149

**Published:** 2021-10-06

**Authors:** Sufang Chen, Wei Guo, Zaixiong Chen, Wenbo Liao, Qiang Fan

**Affiliations:** ^1^ State Key Laboratory of Biocontrol and Guangdong Provincial Key Laboratory of Plant Resources, School of Life Sciences, Sun Yat-Sen University, Guangzhou, China; ^2^ College of Horticulture and Landscape Architecture, Zhongkai University of Agriculture and Engineering, Guangzhou, China; ^3^ Administrative Commission of Danxiashan National Park, Shaoguan, China

**Keywords:** Danxia landform, lithophytic and cave plants, population genomics, Gesneriaceae, founder effect, positive selection

## Abstract

Danxia landform occurring sporadically in southern China is a unique type of petrographic geomorphology. It has nurtured about 400 rare or threatened plant and animal species, whose diversity, endemism, and conservation have called increasing scientific and public attentions. Among them, *Primulina danxiaensis* (W. B. Liao, S. S. Lin, and R. J. Shen) W. B. Liao and K. F. Chung is a tiny perennial grass species recorded only in Mount Danxia, a natural World Heritage Site as part of China’s Danxia. In this study, restriction site-associated DNA sequencing (RAD-seq) was performed to investigate genetic diversity among these 12 populations of *P. danxiaensis*. A total of 432,041 variant sites were detected in 84,779 loci across 94 samples. The expected heterozygosity (*H*_*E*_) ranged from 0.017 to 0.139. Bottleneck signals were detected in most populations, Tajima’s D tests showed that most loci could be under recent positive selection, and one of the six positively selected loci identified by BayeScan was annotated as tRNA^Glu^, which may contribute to the species’ adaptation to shady environment. STRUCTURE analysis and phylogenetic tree showed that the 12 populations of *P. danxiaensis* could be divided into four gene pools (clades) corresponding to their geographic locations, and significant correlation was observed between genetic and geographic distances. Our study demonstrated that *P. danxiaensis* maintained a middle level of genetic diversity and strong population structure; geographic distance could be an important factor limiting gene flow among populations of *P. danxiaensis*, which were only sporadically recorded in Mount Danxia.

## Introduction

Danxia landform, a unique type of petrographic geomorphology sporadically occurred in southern China, is formed from red-colored sandstones and characterized by spectacular red cliffs (Peng 2000). In 2010, nine areas of Danxia landform in subtropical China were inscribed as a Natural World Heritage site (www.worldheritagesite.org), and more than 400 rare or threatened plant and animal species were recorded there (Wang et al., 2008). Named and famous for its spectacular Danxia landform, Mount Danxia is located in northern Shaoguan City, Guangdong Province, China, covering a total area of 292 km^2^ (Peng, 2004). In the past decades, tens of new plant species endemic to Mount Danxia were discovered, such as *Firmiana danxiaensis*, H. H. Hsue and H. S. Kiu, *Primulina danxiaensis* (W. B. Liao, S. S. Lin and R. J. Shen) W. B. Liao and K. F. Chung (Shen et al., 2010), and *Danxiaorchis sinchiana* J. W. Zhai, F. W. Xing, and Z. J. Liu (Zhai et al., 2013).

*Primulina* Hance (Gesneriaceae) comprises ca. 170 species mostly recorded in limestone karst areas (Kong et al., 2017). Species in this genus always occur on steep cliffs and cave entrances, and many of them are found only in a single or micro-areal location, whose current status could be largely threatened under the changeable global climate. Currently, six *Primulina* species were recorded in Mount Danxia. Among them, *P. danxiaensis* and *P. huangjiniana* W. B. Liao, Q. Fan, and C. Y. Huang were endemic to Danxia landform (Huang et al., 2020). During our years of field investigation and protection measurements of the Administrative Commission of Danxiashan National Park, a total of 12 populations of *P. danxiaensis* were currently recorded in Mount Danxia. At the same time, two additional populations were reported in Yongxing, Hunan (Zhang and Yu, 2012) and Ningdou, Jiangxi (Tian et al., 2014). However, a recent study performed by Kong et al. (2017) showed that one individual of *P. danxiaensis* collected from Yongxing, Hunan, did not form a mono clade with other individuals of *P. danxiaensis* collected from Mount Danxia.

Up to date, only several studies have been performed on the population genetics and conservation of *Primulina* species; e.g., Wang et al. (2013) investigated two large populations of *P. tabacum* Hance in Karst caves and found that population D showed considerable genetic divergence while population T demonstrated little genetic structure. Gao et al. (2015) and Wang et al. (2017) investigated phylogenetic relationship among species of the *P. eburnean* (Hance) Yin Z. Wang complex. Their results support the prevalence of allopatric speciation in *Primulina* and highlight the role of geographic isolation in the diversification of *Primulina* species.

The rapid developments of second-generation sequencing technologies provide unprecedented opportunities for genomic studies on these non-model species. The complete genome sequence of *P. huaijiensis* Z. L. Ning and Jing Wang has been reported by Feng et al. (2020). Applying restriction site-associated DNA sequencing (RAD-seq), Wang et al. (2017) found that all the populations of *Primulina juliae* (Hance) Mich. Möller and A. Weber harbored low standing genetic variation and small effective population size, and neutral drift may act as a critical evolutionary driver on population differentiation of the species. In this study, we collected leaf materials from 104 individuals (12 populations) of *P. danxiaensis* under the permission of the Administrative Commission of Danxiashan National Park and performed ddRAD-seq analysis to investigate genetic diversity, diversification, and relationships among these populations. With these effects, we endeavored to provide some valuable information for the protection of these endemic species with extremely small populations.

## Material and Methods

### Study Species

**FIGURE 1:**
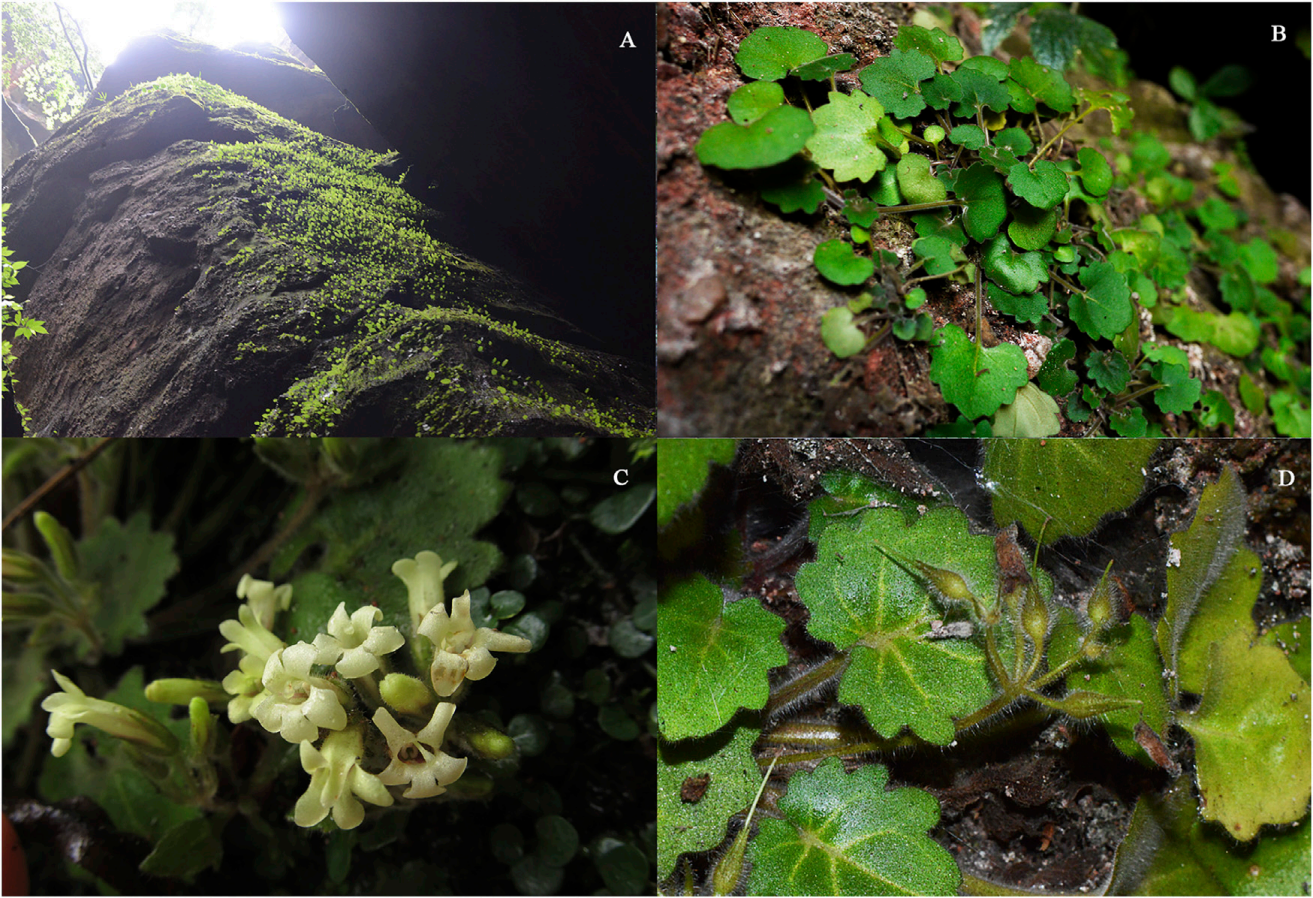
*Primulina danxiaensis* W. B. Liao, S. S. Lin, and R. J. Shen. **(A)** Typical habitat. **(B)** Habit. **(C)** Flower. **(D)** Fruit.

*P. danxiaensis* is a tiny lithophytic perennial herb (2–10 cm in height) occurring on the fissures of large rocks near a shallow pond, wet pit, or shady cave (Figure 1). The species grows in populations that comprised approximately 50–70 individuals and is companied by several other small plants, such as *Boea hygrometrica* (Bunge) R. Br. (Gesneriaceae), *Pteris ensiformis* Burm. f. (Pteridaceae), *Elatostema involucratum* Franch. et Sav. (Urticaceae), *Lindsaea orbiculata* (Lam.) Mett. ex Kuhn (Lindsaeaceae), and *Lophatherum gracile* Brongn. (Poaceae).

### Sample Collection, DNA Extraction, and Restriction Site-Associated DNA Sequencing

**FIGURE 2:**
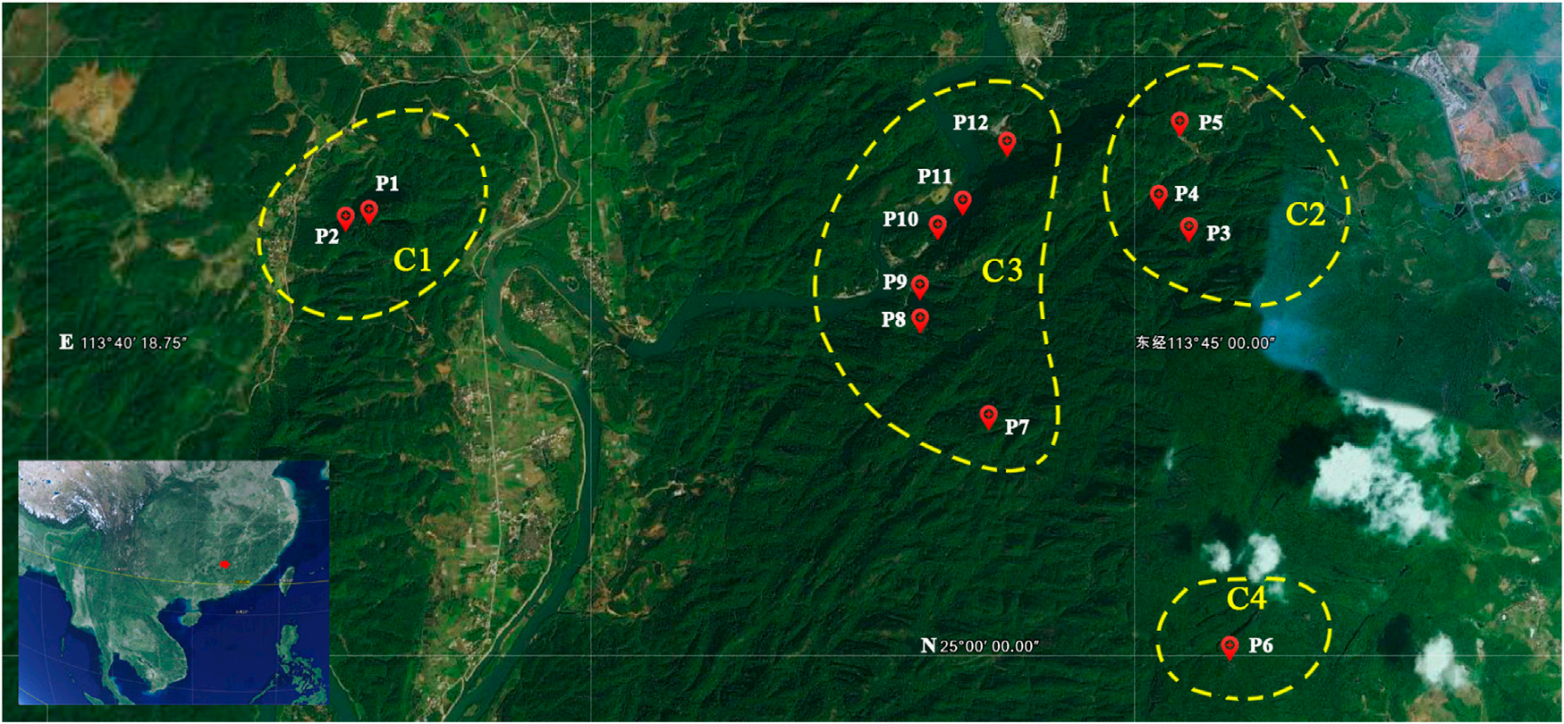
Geographic locations of the 12 populations of *Primulina danxiaensis*. C1–C4: four clades of the phylogenetic tree. P1–P12: population ID for the 12 populations of *P. danxiaensis*.

Fresh leaves were collected for a total of 104 individuals from 12 populations of *P. danxiaensis*. The precise geographic location of each sampled population was determined using a Garmin GPS unit (GPSMAP 62sc, Shanghai, Figure 2 and Table 1). The leaves were immediately dried and stored with silica gel in Ziploc. Genomic DNA was isolated using the modified cetyl trimethylammonium bromide (CTAB) method (Doyle and Doyle, 1987). Genomic DNA was normalized to a concentration of 50 ng/µl, digested with restriction endonuclease *Eco*RI and *Mse*I, ligated to sequencing adaptors and individual barcodes, and amplified by polymerase chain reaction (PCR). Then 16–24 individuals were pulled together and processed into multiplexed RAD libraries following established methods (Baird et al., 2008) and finally sequenced on an Illumina HiSeq 2000 platform (San Diego, CA, United States).

**TABLE 1:** Geographical information and genetic diversity of *Primulina danxiaensis*.

Population	Location	Geographic coordinates	*N*	*H*_*O*_	*H*_*E*_	*F*_*IS*_	Wilcoxon test
P1	Jiemeifeng	25°01′40.36″N, 113°41′42.01″E	8	0.047	0.093	0.134	0.000
P2	Yunkeshan	25°01′38.80″N, 113°41′35.97″E	5	0.019	0.027	0.032	0.002
P3	Xiashanxia	25°01′36.36″N, 113°45′14.14″E	13	0.048	0.047	0.046	0.619
P4	Agongyan	25°01′43.93″N, 113°45′06.36″E	7	0.073	0.139	0.192	0.000
P5	Hukeng	25°02′01.09″N, 113°45′11.77″E	9	0.046	0.101	0.157	0.000
P6	Qingbilong	24°59′58.26″N, 113°45′24.69″E	12	0.016	0.042	0.073	0.000
P7	Huangshakeng	25°00′52.23″N, 113°44′22.33″E	3	0.019	0.017	0.013	0.000
P8	Longxujian	25°01′14.94″N, 113°44′04.62″E	9	0.018	0.039	0.062	0.161
P9	Neishanmen	25°01′22.73″N, 113°44′04.55″E	7	0.019	0.036	0.051	0.000
P10	Yanjiangbu	25°01′36.85″N, 113°44′09.17″E	8	0.019	0.036	0.053	1.000
P11	Jingshiyan	25°01′42.58″N, 113°44′15.63″E	7	0.063	0.128	0.185	0.000
P12	Maweipu	25°01′56.37″N, 113°44′27.12″E	5	0.037	0.061	0.080	0.000

N, the number of individuals; H_O_, observed heterozygosity; H_E_, expected heterozygosity; F_IS_, inbreeding coefficient; Wilcoxon test, a one-tailed Wilcoxon signed-rank test to test the signature of bottleneck

### Internal Transcribed Spacer Sequencing and Phylogenetic Tree Construction

The universal primers ITS_1_ and ITS_4_ were used to amplify the internal transcribed spacer (ITS: containing ITS_1_, 5.8s, and ITS_2_ regions) across 12 individuals of *P. danxiaensis* sampled in Mount Danxia (one individual for each population) and one individual collected from Bingzhou, Hunan. The PCR amplification procedures were performed according to Fan et al. (2014). The obtained sequences were deposited in National Center for Biotechnology Information (NCBI) nucleotide database (accession numbers: MZ723429–MZ723440). According to the phylogenetic tree of *Primulina* (Xu et al., 2019), a total of 215 *Primulina* species were ascribed into four clades (clades A–D), and *P. danxiaensis* was placed in clade D. A total of 96 ITS sequences of *Primulina* (92 in clade D and four in clade C) were downloaded from NCBI nucleotide dataset. With the use of IQtree 1.6.12 (Nguyen et al., 2015), a maximum likelihood tree was then constructed from the 13 ITS sequences of *P. danxiaensis* sequenced in this study and the 96 sequences downloaded from NCBI. The four *Primulina* species in clade C were set as outgroup, and bootstrap support values were estimated from 1,000 replicates.

### Restriction Site-Associated DNA Data Processing

The produced raw data were first filtered with the program *fastp* (Chen et al., 2018) by removing reads containing unknown “N” bases or more than 10% bases with a Q value < 20. Then, the software Stacks 2.55 (Catchen et al., 2013) was used to process the cleaned dataset. At the beginning, the command “process_radtags” was performed to demultiplex individual samples according to their unique barcodes, and 10 samples were removed from further analyses as they contained <500,000 tags. Then, a small dataset containing 15 samples from three populations was used to determine the optimum M (number of mismatches allowed between stacks within individuals) and n (number of mismatches allowed between stacks among individuals), and the command “denovo-map” was performed in which M was set from one to nine and n was set to be equal to M. The optimum M and n were determined to be “4,” as the number of total loci and polymorphic loci increased to a platform when M = n = 4. At last, the command “denovo-map” was performed to process all the 94 samples with the optimum M and n values, and the command “populations” was used to filter the results by setting “--min-maf 0.05 max-obs-het = 0.8.”

### The Detection of Loci Under Selection

To detect non-neutral loci under selection, Tajima’s D was calculated using VCFtools (Danecek et al., 2011), in which the window size was set as 3,000 bp. Loci with Tajima’s D value <−1.795 or >2.052 were deemed as non-neutral (Tajima, 1989; Liu et al., 2020). According to Tajima’s D value, the dataset was split into two datasets: 1) neutral dataset (−1.795 < Tajima’s D value <2.052); and 2) non-neutral dataset (Tajima’s D value <−1.795 or >2.052). The software PGDSpider v2.1.1.5 (Lischer and Excoffier, 2012) was used to format conversion in subsequent analyses. For the non-neutral dataset, the third single-nucleotide polymorphism (SNP) in each locus was extracted from the Variant Calling File (VCF) and transformed to BayeScan format; then BayeScan v.2.1 was used to detect loci under selection with default parameters (Foll and Gaggiotti, 2008).

### Population Data Analyses

For the neutral and non-neutral datasets, signatures of bottlenecks were detected using the program Bottleneck 1.2.02 (Piry et al., 1999), in which a one-tailed Wilcoxon signed-rank test was performed under the infinite alleles model (IAM). Principal coordinates analysis (PCoA) was performed with Plink v1.90 (Chang et al., 2015), and python script and R 4.1.0 (R Development Core Team, 2011) were used to draw the scatter diagram. Then Bayesian cluster analysis was conducted with the software STRUCTURE v2.3.4 (Pritchard et al., 2000). The number of groups (*K*) was set from 2 to 6, and the optimal *K* was determined by the online Structure Harvester (http://taylor0.biology.ucla.edu/structureHarvester/). A phylogenetic tree based on maximum likelihood method was constructed with the IQtree, setting “-m MFC+ASC–alrt 2000.”

These 12 populations were then divided into groups according to STRUCTURE analysis; the software Arlequin 3.5.2.2 (Excoffier and Lischer, 2010) was used to perform analysis of molecular variance (AMOVA) among groups, among populations within groups, and within populations.

Pairwise *F*_*ST*_ was calculated based on Kimura 2P method, and *N*_*M*_ was estimated based on the formula *N*_*M*_ = 1/(4 * *F*_*ST*_ + 1). The Mantel test was performed with GenAlEx 6.5 (Peakall1, 2012) to calculate correlation between genetic distance *F*_*ST*_/(1 − *F*_*ST*_) and geographic distance (ln) (Diniz et al., 2013).

## Results

### Phylogenetic Position of *Primulina danxiaensis*

**FIGURE 3:**
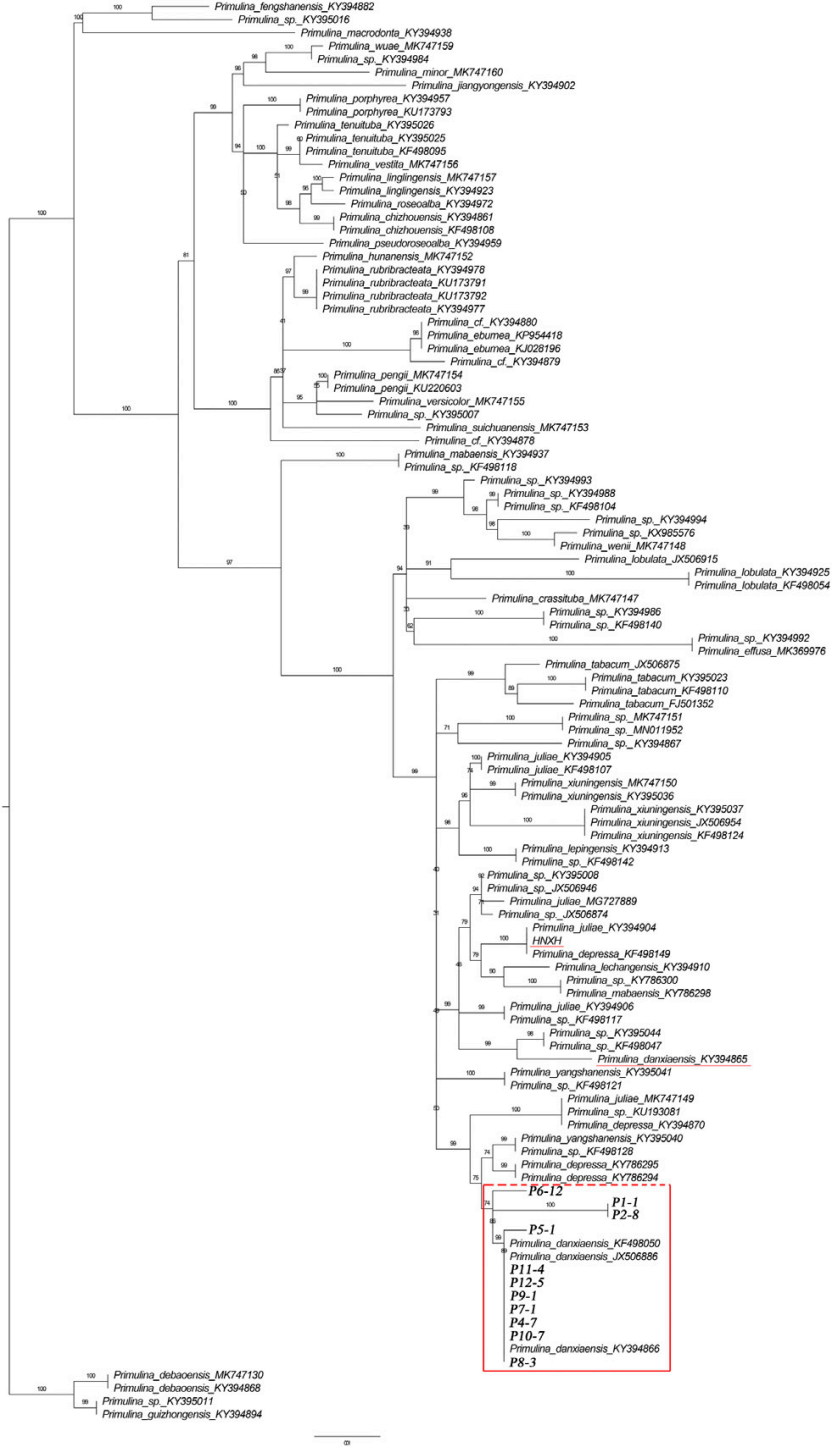
Phylogenetic tree of 109 *Primulina* species constructed from ITS sequences. P1-1–P12-5: *Primulina danxiaensis* sampled from Mount Danxia. HNXH: *P. danxiaensis* sampled from Hunan.

The phylogenetic tree constructed from ITS sequences showed that the 12 individuals of *P. danxiaensis* collected from Mount Danxia clustered together with three individuals of *P. danxiaensis* downloaded from NCBI website and formed a monogroup with a weak support value (PP: 74%). Two samples of *P. danxiaensis* (one was downloaded from NCBI and the other was sampled from Hunan) were placed in two other positions of the phylogenetic tree (Figure 3).

### Restriction Site-Associated DNA Sequencing and Genetic Diversity

A total of 1,127,173,204 short reads were produced for the 104 samples. With the use of the program “process_radtags,” the number of tags retained for each samples ranged from 25,656 to 31,249,373 (averagely 3,416,629), and 10 samples with tags less than 500,000 were removed from further analyses. By applying the program “*de novo* map” and the optimum value of M = n = 4, a total of 747,038 loci containing 1,365,868 variant sites were produced across the 94 samples. After filtration with the program “populations,” 432,041 variant sites in 84,779 loci were retained in this dataset (Dataset T).

The observed heterozygosity (*H*_*O*_) for the 12 populations ranged from 0.016 (P6) to 0.073 (P4) with an average value of 0.035 ± 0.019, the expected heterozygosity (*H*_*E*_) ranged from 0.017 (P7) to 0.139 (P4) with an average value of 0.063 ± 0.039, and the inbreeding coefficient (*F*_*IS*_) ranged from 0.013 to 0.192 with an average value of 0.089 ± 0.059 (Table 1).

### The Scan of Non-Neutral Loci

**FIGURE 4:**
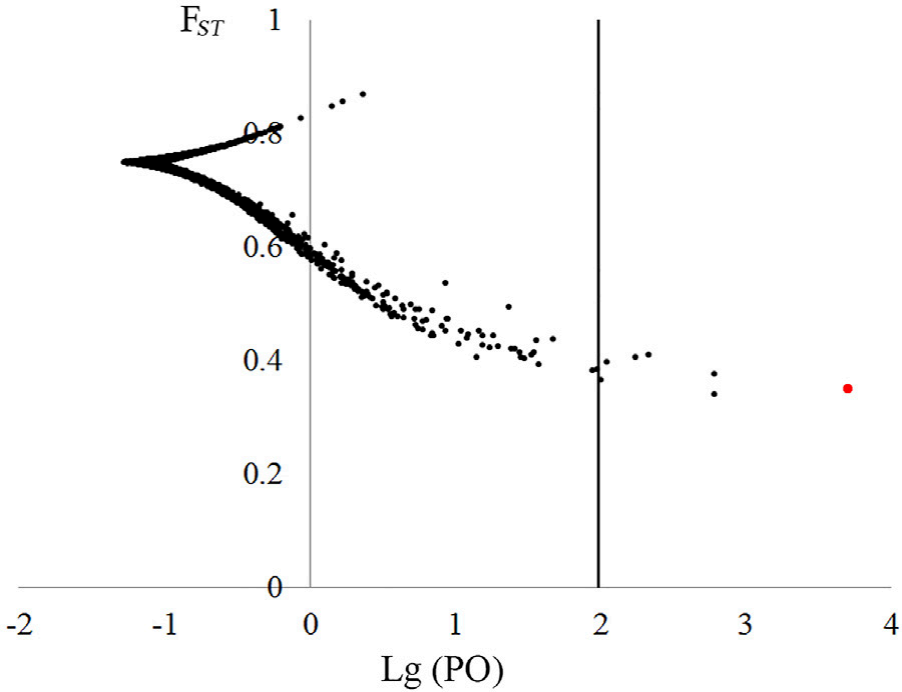
Plot of *F*_*ST*_ values and log10(PO) for 40,751 non-neutral loci based on BayeScan outlier test. Loci in the right of the dashed line (log10[PO] = 2) were deemed as under selection with a posterior probability >0.99.

For the original 84,779 loci across 94 samples, Tajima’s D values ranged from −0.846 to 5.166, and two datasets were constructed: 1) neural dataset containing 34,518 putative neutral loci (Tajima’s D value ranged from −0.846 to 2.052) and 2) non-neutral dataset consisted of 50,261 loci with Tajima’s D value > 2.052. Based on the non-neutral dataset, BayeScan analysis showed that seven loci could be under positive selection with high possibility (*p* > 0.99, Figure 4). A BLASTn search against NCBI nucleotide database showed six loci had no significant hits, and one showed high similarity with chroloplast gene tRNA^Glu^ (E-value: 0.0).

### Analysis of Population Genetic Structure

**FIGURE 5:**
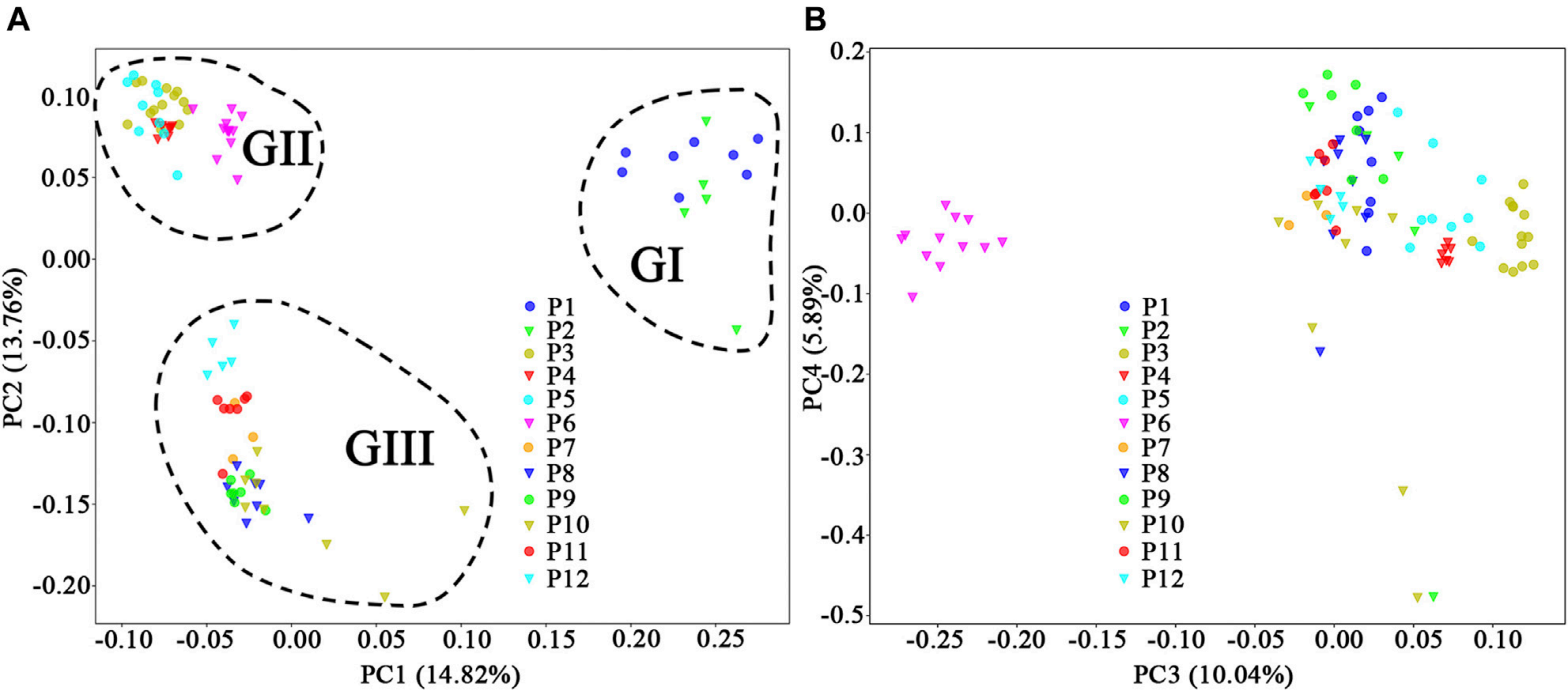
Principal coordinates analysis of individual genotypes obtained from 12 populations of *Primulina danxiaensis* based on the natural dataset. **(A)** Plot of PC1 and PC2. **(B)** Plot of PC3 and PC4. P1–P12: population ID for the 12 populations of *P. danxiaensis*.

Based on the neutral dataset, signals of bottleneck were detected in nine of the 12 populations (Table 1). PCoA showed that the first four coordinates (PC_1_–PC_4_) explained 14.82, 13.76, 10.04, and 5.89% of the total variation, and the 94 individuals were divided into three groups according to PC_1_ and PC_2_ (Figure 5A): GI containing all the individuals from populations P1 and P2; GII including all the individuals of populations P3, P4, P5, and P6; and GIII comprising all the individuals of the rest of the populations. The third coordinate (PC_3_) separated population P6 from all others (Figure 5B). For the non-neutral dataset, PLINK failed to extract eigenvectors.

**FIGURE 6:**
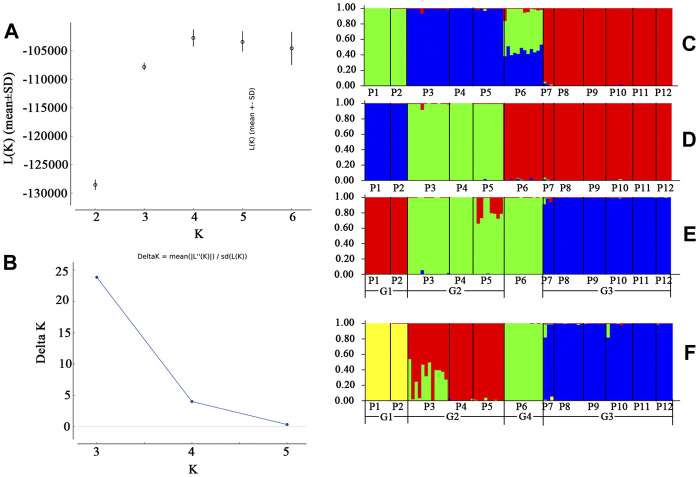
Unrooted maximum likelihood phylogenetic tree constructed from the 94 individuals of *Primulina danxiaensis* based on dataset T. P1–P12: population ID for the 12 populations of *P. danxiaensis*.

**FIGURE 7:**
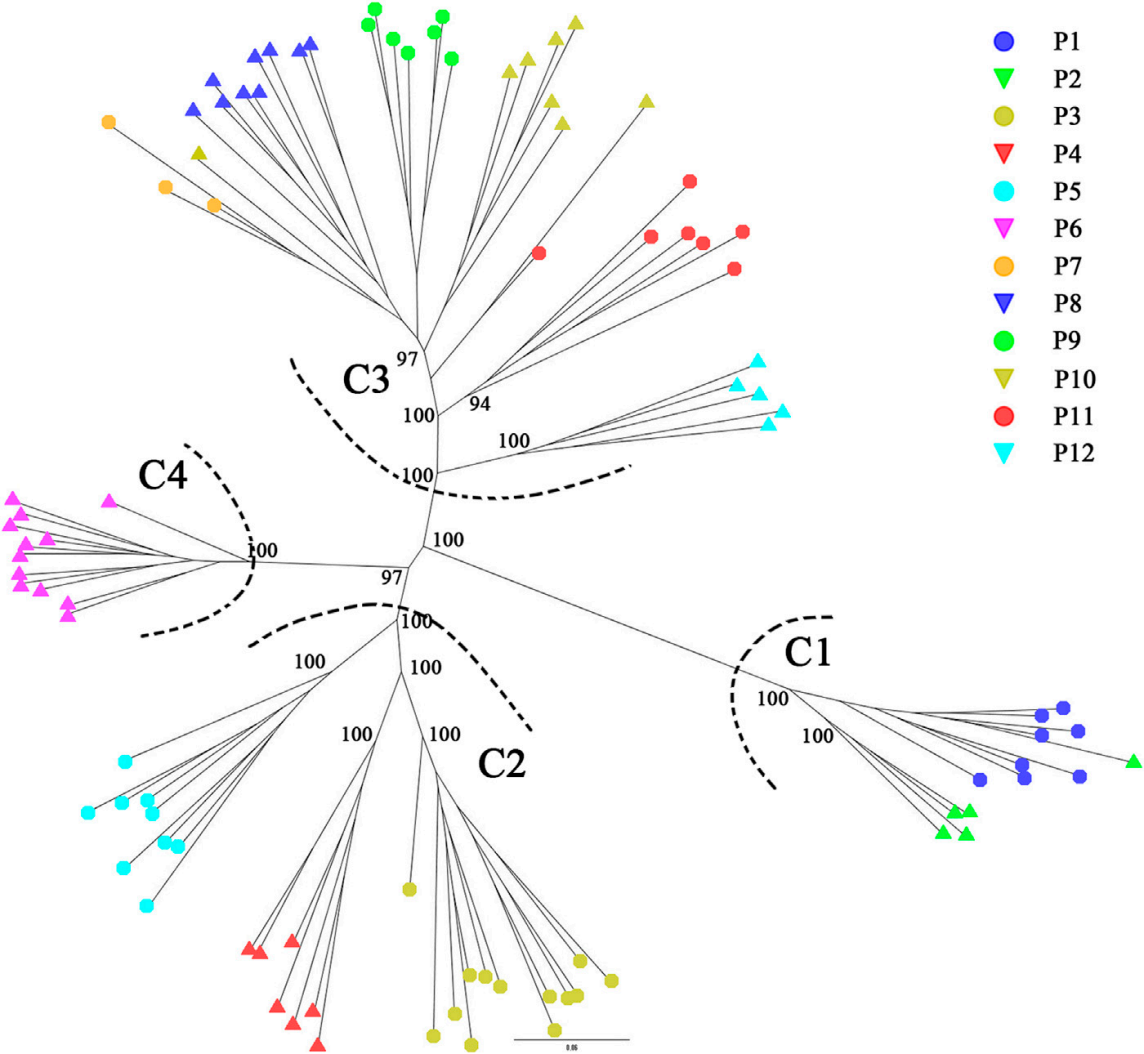
STRUCTURE assignment results of the 94 individuals from 12 populations for *K* = 3, *K* = 4, and *K* = 5. Different colors represent different gene pools. P1–P12: population ID for the 12 populations of *Primulina danxiaensis*. C1–C4: four clades assigned in phylogenetic tree.

STRUCTURE analysis was performed across 10,573 loci. Results based on ΔK and mean Ln P(*K*) indicated optimal values of 3 and 4, respectively (Figures 6A,B). At *K* = 3, the patterns of genetic clustering were not completely converged, and the 94 individuals of the 12 populations were divided into three gene pools (G1–G3), which was in accordance with the three groups (GI–GIII) divided by PC_1_ and PC_2_ in PCoA, though population P6 was assigned to mixed gene pools from G1 and G2 in three iterations, gene pool G3 in two iterations, and G2 in one iteration (Figures 6C–E). At *K* = 4, all the individuals of P6 were assigned to a new gene pool G4 (Figure 6F). The phylogenetic tree constructed from the neutral dataset showed that all the 94 individuals of *P. danxiaensis* were clustered into four main clades (C1–C4, Figure 7) in accordance with the four gene pools in STRUCTURE analysis when *K* = 4.

As the 94 individuals were assigned into four groups according to STRUCTURE analysis, AMOVA (Table 2) showed that the genetic diversity of *P. danxiaensis* is primarily maintained within populations (56.73%, *F*_*ST*_ = 0.433, *p* < 0.001), though a significant proportion of genetic variation was also observed among groups (31.46%, *F*_*CT*_ = 0.316, *p* < 0.001).

**TABLE 2:** Analysis of molecular variance (AMOVA) for the 12 populations of *Primulina danxiaensis*.

Source of variation	Sum of squares	Variance components	Percentage of variation	F-statistics
Among groups	993.155	12.210	31.46	*F*_*CT*_ = 0.316^*^
Among populations within groups	436.188	4.584	11.81	*F*_*SC*_ = 0.172^*^
Within populations	1,805.073	22.013	56.73	*F*_*ST*_ = 0.433^*^

^*^ *p*-value < 0.001.

**FIGURE 8:**
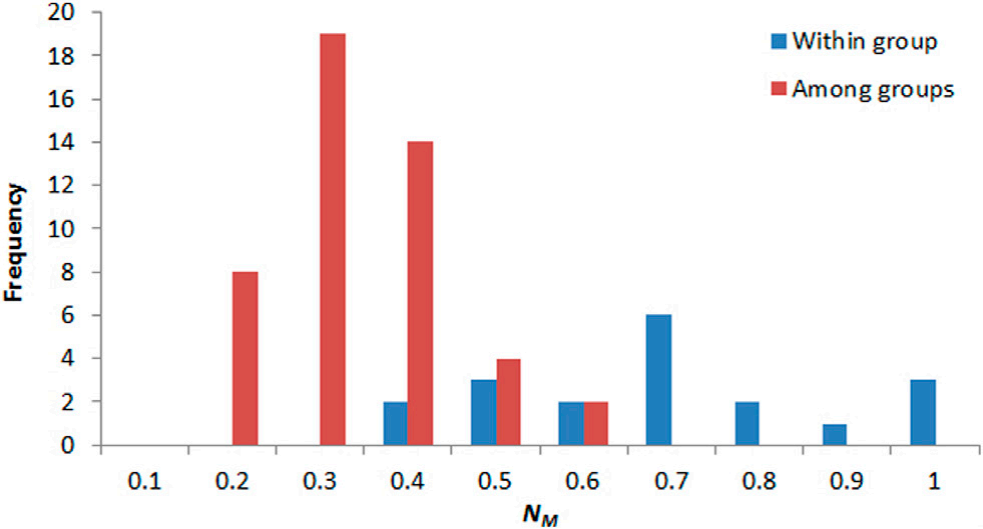
Frequency distribution of pairwise gene flow between populations within group (red) and among groups (blue).

**FIGURE 9:**
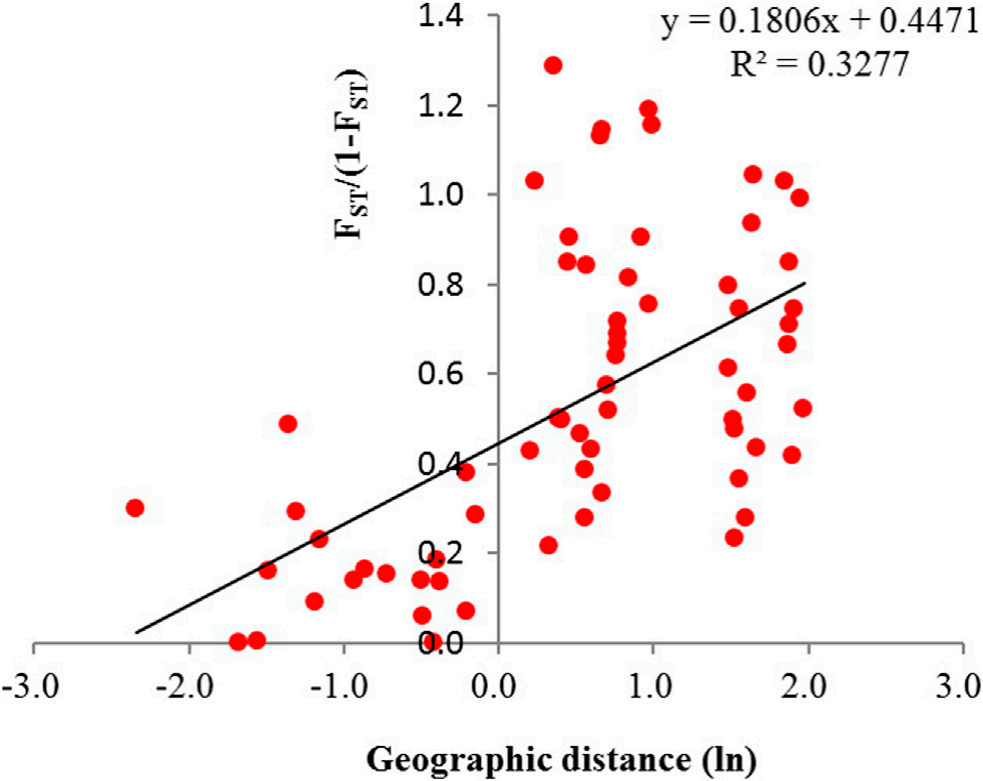
Relationship between pairwise *F*_*ST*_/(1 − *F*_*ST*_) and the geographic distance (ln) among the 12 populations of *Primulina danxiaensis* (*r* = 0.572, *p* < 0.001).

The pairwise *F*_*ST*_ between populations ranged from 0 to 1.287, and *N*_*M*_ ranged from 0.163 to 1 (Supplementary Table S1). *N*_*M*_ between populations in the same group ranged from 0.3 to 1 and centered in 0.6–0.7, and *N*_*M*_ between populations in different groups ranged from 0.1 to 0.6 and centered in 0.2–0.3 (Figure 8). Significant correction was found between genetic differentiation (*F*_*ST*_/(1 − *F*_*ST*_)) and geographic distance (ln): *r* = 0.572, *p* < 0.001 (Figure 9).

## Discussion

### The Monophyly of *Primulina danxiaensis*

Since *P. danxiaensis* was first found in Mount Danxia of Guangdong (China), two more populations were successively reported in other Danxia landforms of Jiangxi and Hunan (China). It is interesting to know whether these populations are the same species or whether they are a monophyletic group in the phylogenetic tree. Our study based on ITS sequences of 105 *Primulina* samples showed that all the individuals of *P. danxiaensis* sampled from Mount Danxia formed a monophyletic group, while two other individuals collected from Hunan were placed in two different places, indicating that all the 12 populations sampled from Mount Danxia should be the same species *P. danxiaensis*, while those sampled from the other Danxia landform could have evolved independently with morphological characters much similar to those of *P. danxiaensis*.

### Median Level of Genetic Diversity Under Strong Environment Pressure

The genetic diversity of *P. danxiaensis* (*H*_*E*_: 0.063 ± 0.039) is much lower than that of the tree species *Cryptomeria japonica* subsp. *sinensis* (Miq.) P.D.Sell sampled in southern China and Japan (*H*_*E*_: 0.253 ± 0.002, RAD-seq data in Cai et al. (2020), close to threaten shrub species *Rhododendron cyanocarpum* Franch. & W.W.Sm. (*H*_*E*_: 0.068 ± 0.002, RAD-seq data in Liu et al. (2020), and higher than the congener species *P. juliae* mainly recorded on Karst and Danxia landforms located in the Nanling Mountains (*H*_*E*_: 0.047 ± 0.020; RAD-seq in Wang et al. (2017). It is inspiring, as *P. danxiaensis* is narrowly confined in Mount Danxia, and only tens of scattered populations have been recorded to date.

Bottleneck analyses revealed that most populations of *P. danxiaensis* have experienced founder effects. This phenomenon was also observed in many populations of *P. juliae* (Wang et al., 2017). The neutral test of Tajima’s D showed that more than half of the detected loci possessed a Tajima’s D value > 2.052, suggesting that many loci could be under positive selection or reflect a recent population contraction. The shallow soil surface of naked rock containing low levels of nutrition could have exerted great pressure for the survival of species living there. BayeScan analysis showed that one locus under positive selection was annotated as tRNA^Glu^, a dual-function molecule participating both in protein and in 5-aminolevulinic acid (ALA) and, hence, chlorophyll biosynthesis (Stange-Thomann et al., 1994). Strong positive selection detected on the gene may affect the biosynthesis of chlorophyll and further facilitates light absorbance for the species that survives in a poor light environment.

### Strong Genetic Differentiation Within Short Distances

Though within a very short geographic distance (no more than 8 km between any two populations), *P. danxiaensis* demonstrated a strong population structure, in which individuals were divided into four gene pools (clades) corresponding to their geographic locations (Figures 2, 3). AMOVA showed that 31.46% genetic variations were observed among groups. This high population differentiation was also observed in *P. tabacum* in a single cave (Wang et al., 2013), *Kunzea pulchella* (Lindl.) A. S. George endemic to granite landform of Western Australia (Tapper et al., 2014).

Genetic differences in *P. danxiaensis* among groups could have developed through limited gene flow. *N*_*M*_ values between populations of *P. danxiaensis* were low in most cases (*N*_*M*_ < 1), especially between populations in different groups (Figure 8), and significant correlation was found between pairwise *F*_*ST*_ and geographic distance, indicating that geographic distance could be an important factor limiting the level of gene flow between populations of *P. danxiaensis* in short geographic distance. Some other studies also found that isolation by distance can occur over short distances in alpine species, such as *Peucedanum multivittatum* Maxim. across a snowmelt gradient <1 km long (r = 0.429, *p* = 0.009; Hirao and Kudo, 2004).

The pollens of *Primulina* species were dispersed by bees, while its tiny seeds can be dispersed by wind. The value of *F*_*IS*_ was estimated to be 0.089 ± 0.059 (Table 1), suggesting that *P. danxiaensis* is a weakly inbreeding species. However, the population of *P. danxiaensis* was very small (always in a single exposed rock) and scarce, and the chance for the pollens or seeds to be dispersed from one population to another could be very low, which may seriously affect gene flow between populations, even in short distance.

### Conservation of *Primulina danxiaensis*

Our study showed that *P. danxiaensis* is a plant species with extremely small populations (PSESP) demonstrating strong genetic structure, which is different from some tree species such as *F. danxiaensis*, a PSESP endemic to Danxia landform with moderate genetic diversity (*H*_*E*_ = 0.364 ± 0.019) and weak genetic structure in Mount Danxia (SSR data, Chen et al., 2014). It was obvious that different measures should be taken for the conservation of these two PSESP species. For *P. danxiaensis*, it only had tens of small isolated populations, and measures should be taken to conserve each extant population of the species. Furthermore, scientific studies should also pay attention to the optimal habitats for the survival of the species, and seeds of the species can be manually collected and dispersed to some other potential habitats so that new populations could possibly be established.

## Data Availability

RAD raw data was depostied to the NCBI SRA database with bioproject ID PRJNA736829, in which 94 biosamples and 94 SRA files was uploaded. ITS sequences for *P. danxiaensis* were deposited in GenBank with accession number MZ723429-MZ723440.
