# Supplementary material for: Strong Genetic Structure Observed in Primulina danxiaensis, a Small Herb Endemic to Mount Danxia With Extremely Small Populations
Source: Front Genet. 2021 Oct 6;12:722149. doi: 10.3389/fgene.2021.722149 (PMC8526925; doi:10.3389/fgene.2021.722149)
Supplement: Supplementary file 1 [file Table1.DOCX]

Table S1 Pairwise *N_M_* (above the diagonal) and pairwise genetic differentiation (*F_ST_*) (below the diagonal) of the 12 populations of *P. danxiaensis*.

|  | P1 | P2 | P3 | P4 | P5 | P6 | P7 | P8 | P9 | P10 | P11 | P12 |
| --- | --- | --- | --- | --- | --- | --- | --- | --- | --- | --- | --- | --- |
| P1 | --- | 1.000 | 0.228 | 0.195 | 0.273 | 0.201 | 0.310 | 0.289 | 0.238 | 0.334 | 0.251 | 0.211 |
| P2 | 0.000 | --- | 0.251 | 0.261 | 0.375 | 0.324 | 0.193 | 0.515 | 0.344 | 0.407 | 0.474 | 0.365 |
| P3 | 0.849 | 0.744 | --- | 0.339 | 0.520 | 0.195 | 0.349 | 0.259 | 0.272 | 0.303 | 0.367 | 0.333 |
| P4 | 1.031 | 0.709 | 0.488 | --- | 0.462 | 0.163 | 0.335 | 0.179 | 0.181 | 0.229 | 0.227 | 0.369 |
| P5 | 0.666 | 0.417 | 0.230 | 0.291 | --- | 0.216 | 0.472 | 0.266 | 0.281 | 0.427 | 0.394 | 0.538 |
| P6 | 0.992 | 0.521 | 1.032 | 1.287 | 0.906 | --- | 0.326 | 0.174 | 0.178 | 0.249 | 0.216 | 0.234 |
| P7 | 0.557 | 1.045 | 0.466 | 0.496 | 0.280 | 0.517 | --- | 0.808 | 0.577 | 0.648 | 1.000 | 0.784 |
| P8 | 0.614 | 0.235 | 0.717 | 1.146 | 0.690 | 1.190 | 0.059 | --- | 0.454 | 0.732 | 0.618 | 0.468 |
| P9 | 0.799 | 0.477 | 0.669 | 1.132 | 0.639 | 1.156 | 0.183 | 0.301 | --- | 0.609 | 0.605 | 0.398 |
| P10 | 0.499 | 0.365 | 0.574 | 0.843 | 0.335 | 0.755 | 0.136 | 0.091 | 0.160 | --- | 0.992 | 0.643 |
| P11 | 0.744 | 0.277 | 0.431 | 0.850 | 0.385 | 0.906 | 0.000 | 0.154 | 0.164 | 0.002 | --- | 0.645 |
| P12 | 0.936 | 0.436 | 0.500 | 0.427 | 0.215 | 0.816 | 0.069 | 0.285 | 0.378 | 0.139 | 0.138 | --- |
